# Préoccupations de carrière chez les médecins de travail des groupements de Médecine de travail en Tunisie

**DOI:** 10.11604/pamj.2017.27.50.11431

**Published:** 2017-05-19

**Authors:** Irtyah Merchaoui, Asma Chouchène, Ines Bouanène, Néila Chaari, Wassim Zrafi, Adnène Henchi, Mohamed Akrout, Charfeddine Amri

**Affiliations:** 1 Service de Médecine de Travail et de Pathologie Professionnelle, Centre Hospitalo-Universitaire de Monastir,Tunisie; 2 Service de Médecine Communautaire, Centre Hospitalo-Universitaire de Monastir,Tunisie

**Keywords:** Satisfaction au travail, carrière, salaire, médecins de travail, Job satisfaction, occupational medicine, Occupational Health Services, occupational physicians, Tunisia

## Abstract

**Introduction:**

L'insatisfaction de carrière des médecins de travail (MT) peut influencer leur performance et la qualité des prestations fournies. L'objectif de notre étude est d'évaluer la satisfaction au travail des MT du terrain de l'ensemble des Groupements de médecine de travail (GMT) de la Tunisie et préciser les facteurs déterminants.

**Méthodes:**

Il s'agit d'une étude nationale transversale portant sur les MT de 22 GMT, basée sur le questionnaire, validé SAPHORA JOB.

**Résultats:**

58% des MT des GMT étaient insatisfaits de leur carrière. La satisfaction de carrière était statistiquement influencée par le nombre d'entreprises en charge (p=0,016), l'organisation du travail (p=0,010),le ressenti du métier (p=0,011),le salaire (p‹10-3) et l'information sur la réglementation en vigueur (p=0,047).

**Conclusion:**

L'homogénéisation des grilles salariales et des échelons de carrière des MT des GMT basée sur une révision des textes législatifs est indiquée. L'amélioration de l'organisation et des conditions de travail peut permettre un épanouissement au travail et une amélioration des prestations.

## Abstract (en)

**Introduction:**

Career dissatisfaction among occupational physicians (OP) may affect their performance and the quality of healthcare services provided. Our study aims to assess the job satisfaction of OP serving on the Institutes for Occupational Safety and Health(IOSH) in Tunisia and to identify the determinant factors.

**Methodology:**

We conducted a national cross-sectional validated SAPHORA JOB survey among all the OP serving on 22 IOSH.

**Results:**

58% of OP serving on the IOSH were dissatisfied with their career. Career satisfaction was statistically influenced by the number of healthcare facilities for which they were responsible (p=0.016), the work organisation (p=0.010), the work impact (p=0.011), the salary (p‹10-3) and knowledge on current regulation (p=0.047).

**Conclusion:**

The standardisation of pay scale and career ladders for OP serving on the IOSH based on a revision of legislative texts is recommended. Improving organization and working conditions may allow success in the workplace and benefit enhancement.

## Introduction

La satisfaction au travail est un concept qui permet de cerner les attitudes des employés face à leur travail. Il s'agit d'une évaluation subjective élaborée par un individu des aspects clefs de son travail, en fonction de ses attentes, ses expériences et ses valeurs. La focalisation de plusieurs études dans la littérature sur ce sujet a permis d'établir des moyens objectifs d'évaluation à l'aide d'instruments de mesures multidimensionnels (1) [1]. En fait, la définition de la satisfaction au travail a beaucoup évolué au fil des années. Une revue de la littérature menée par la Comité de Coordination de l'Evaluation Clinique et de la Qualité en Aquitaine (CCECQA) et mise à jour en 2011, portant sur la révision de la définition de la satisfaction au travail, présente ce concept comme « une perception instable » qui se modifie tout au long de la vie du salarié (1) [2]. L'étude de la satisfaction au travail chez le personnel de la santé a fait l'objet de nombreux travaux dont les résultats ont montré une grande divergence entre les différents pays [3]. Cela parait être en rapport avec des différences aussi bien dans les conditions de travail que dans les différences culturelles, dans l'expérience et dans les attentes face au travail entre les pays. Sur le plan national, bien que la satisfaction au travail soit l'un des concepts clefs des ressources humaines, le sujet n'a pas été fréquemment abordé par les études de recherches chez le cadre médical en général, ni chez les médecins de travail du terrain en particulier. En Tunisie, c'est grâce au décret beylical du 20 Septembre 1955 relatif à l'Inspection Médicale du Travail que la Médecine du Travail a vu le jour. Ensuite, il a fallu attendre le 30 Avril 1966, date d'apparition du Code du Travail pour avoir une référence en matière de législation relative à l'organisation des services de Médecine du Travail. Actuellement, les prestations de Médecine de travail dans les entreprises tunisiennes sont essentiellement assurées par des organismes dits « groupements de Médecine de Travail », qui gèrent la répartition et la coordination des Médecins de travail du terrain dans les gouvernorats où ils sont territorialement compétents. Le Médecin de travail du groupement se déplace ainsi d'une entreprise à une autre selon un emploi du temps hebdomadaire fourni par le Médecin coordinateur du groupement [4]. Malgré cette relative ancienneté de la Médecine du Travail en Tunisie, le nombre croissant d'entreprises et de salariés, le rôle indiscutable des Médecins du Travail dans la prévention des risques professionnels et des maladies d'ordre général, très peu d'études se sont intéressées à l'épanouissement des Médecins du Travail des groupements dans l'accomplissement de leurs prestations. Notre étude a pour but d'évaluer la satisfaction de la carrière au travail des Médecins du Travail du terrain en Tunisie et d'en identifier les déterminants.

## Méthodes

Il s'agit d'une étude exhaustive transversale auprès de l'ensemble des 136 Médecins du travail de terrain, œuvrant au sein de 22 Groupements de Médecine du Travail, en exercice durant la période allant de Mai 2010 à Février 2011 (Tableau 1). Le seul critère d'exclusion a été l'ancienneté professionnelle moindre d'une année. Notre étude s'est déroulée durant la période allant de Mai 2010 à Février 2011.Elle s'est basée sur un questionnaire anonyme, auto-administré, inspiré du questionnaire validé « SAPHORA-Job^®^ » [5,6] qui explore la satisfaction Médecins du Travail de terrain dans 7 dimensions dont celles de la carrière et du salaire. Ces dimensions sont exprimées par sept indicateurs de satisfaction : les indicateurs « Travail » ; « Carrière » ; « Salaire » ; « Encadrement » ; « Réglementation » ; « Reconnaissance » ; «Etablissement ». La collecte des données s'est réalisée à travers l'envoi par courrier postal, par Fax et par courrier électronique. Tous les Médecins coordinateurs de tous les GMT ont été préalablement contactés afin d'expliquer les objectifs et de les assurer de confidentialité des données collectées.

**Tableau 1:** Répartition des Médecins du Travail par Groupement

GMT*	N** de Médecins	N** d’Entreprises	N** de Salariés
Tunis	8	469	31082
Ariana	5	290	16173
Mannouba	5	297	16093
Ben Arous	9	974	34343
Nabeul	13	1191	60749
Zaghouan	5	357	20479
Bizerte	7	545	36897
Béja	3	170	10169
Jendouba	3	227	7627
Le Kef	1	171	3898
Siliana	3	663	6823
Kairouan	2	104	6250
Kasserine	2	101	4275
Sidi Bouzid	1	147	2702
Sousse	16	904	50321
Monastir	13	1087	55016
Mahdia	5	304	11669
Sfax	25	3763	66862
Gafsa	1	99	5390
Torzeur	1	206	3532
Gabes	5	372	12368
Medenine	3	423	16243
Total	136	12864	478961

* Groupements de Médecine de Travail

** Nombre

**Analyse et traitement des données:** Le calcul des scores des indicateurs s'est basé sur la formule générale de calcul de scores de satisfaction suivante [**6**]. Score=(20*Σ(valeurs des items répondus)-N)/N; N : est le nombre d'items répondus par indicateur. La valeur de chaque item varie de 1 à 6 (pas du tout satisfait à tout à fait satisfait). Le score global de satisfaction n'a pas été calculé, l'objectif étant d'explorer les dimensions de satisfaction de la carrière. L'analyse uni-variée a pris en considération les déterminants socio-professionnels et les autres scores relatifs aux conditions d'exercice. Dans l'analyse multi variée, une régression linéaire multiple a été effectuée pour les scores et les indicateurs de satisfaction. Une équation du modèle prédictif de la variation de la satisfaction de carrière a été ainsi créée. L'inclusion des variables indépendantes dans les modèles de régression a été effectuée lorsque le degré de signification était < 0.25. Le seuil de signification des tests statistiques a été fixé à 0,05.

## Résultats

Le taux de participation des GMT à notre enquête était de 59%, celui des Médecins du travail était de 52%. Le sex-ratio était de 0,46, l'âge moyen était de 40,3 ans ± 5 ans avec des extrêmes allant de 32 à 51 ans. L'ancienneté de nos médecins était supérieure à 10 ans dans le quart des cas et à 20 ans chez 4.5 % d'entre eux Tableau 1. La médiane du nombre d'entreprises prises en charge par Médecin du Travail était de 30 avec des extrêmes allant de 6 à 380 entreprises par Médecin .Dix-neuf pour cent de nos médecins interrogés avaient moins de 20 entreprises en charge. L'effectif moyen de salariés pris en charge par année et par Médecin de Travail de terrain était de 2942 ± 480 tous secteurs d'activité et groupements confondus avec des extrêmes allant de 1700 à 4000 salariés. Cinquante-huit pour cent des Médecins du Travail de terrain interrogés était insatisfaits de leur carrière professionnelle (58,2%). Leur score global moyen de satisfaction de carrière était de 43,4±19. L'étude analytique uni-variée a montré qu'aucune des caractéristiques socio-professionnelles n'influençait de façon significative la satisfaction de carrière des Médecins de travail de terrain Tableau 2. En revanche, les scores des indicateurs de satisfaction de l'« organisation du travail » (p‹10-3), de la « Nature de travail » (p=0,007), du « Ressenti du métier » (p‹10-3 ), du « Salaire » (p‹10-3 ), de l'« Encadrement » (p‹10-3 ) et de l'« information sur la réglementation » (p‹10-3 ) ainsi que la satisfaction de la relation (p‹10-3 ) et de la communication (p‹10-3 ) avec le groupement ou de la relation avec les entreprises (p‹10-3 ) augmentaient significativement avec la satisfaction de carrière Tableau 3. Le modèle final de régression multivariée a montré que la satisfaction de « Carrière » était statistiquement influencée par les co-variables suivantes à savoir : le nombre d'entreprises attribuées au Médecin de travail (p=0,016), la satisfaction vis-à-vis de l'organisation du travail (p=0,010), la satisfaction du ressenti du métier (p=0,011), la satisfaction du salaire (p< ‹10-3) et de l'information sur la réglementation en vigueur (p=0,047). (Tableau IV) Ce Modèle expliquait 66,4% de la fluctuation de la satisfaction de carrière. La variable satisfaction de salaire avait le coefficient le plus élevé (0,316). Donc si tous les autres prédicteurs sont constants, chaque augmentation de la satisfaction de carrière d'une unité de plus, est associée à une augmentation de 0,316 x score de satisfaction du salaire. (Tableau IV) Le modèle final prédictif de la satisfaction de carrière des Médecins de travail des groupements de Médecine de Travail est le suivant : Satisfaction de carrière prédite =-5,965+0,067 X1+0,258 X2+0,208 X3+0,316 X4+0,154 X5 X1 : Nombre d´entreprises, X2 : satisfaction de l'organisation au travail, X3 : satisfaction du ressenti du métier, X4 : satisfaction du salaire, X5 : satisfaction de l'information sur la réglementation Tableau 4.

**Tableau 2:** Répartition de la population d’étude selon les caractéristiques socio-professionnelles

Caractéristiques socio-professionnelles des Médecins des Groupements	N	%
Genre		
Homme	21	31.3
Femme	46	68,7
Formation diplômante en Médecine du Travail	63	94
Choix délibéré de la discipline	34	50,7
Originaire de la région	45	67,2
Statut CDI	50	74,6
Mode d’activité mono-sectorielle	13	19,4

**Tableau 3:** Variables influençant la satisfaction de carrière

Variables	Score carrière	Β^+^	p
**Socio-professionnelles**			
Genre			0,107
Homme	46,67		
Femme	41,93		
Ancienneté		0,15	0,20
Formation diplômante en	43,45		0,74
Médecine du Travail			
Choix délibéré de la discipline	48,24		0,06
Originaire de la région	42,22		0,64
Statut CDI	44,86		0,40
Nombre d’entreprises en charge		0,22	0,09
Nombre de salariés en charge		-0,09	0,5
**Scores des autres items de satisfaction**			
L’organisation du travail		0,55	**˂ 10^-3^**
Nature de travail		0,34	**0,007**
Ressenti du métier		0,5	**˂ 10^-3^**
Salaire		0,65	**˂ 10^-3^**
Encadrement		0,59	**˂ 10^-3^**
information réglementation		0,60	**˂ 10^-3^**
**Etablissement GMT**			
Relation		0,56	**˂ 10^-3^**
Communication		0,45	**˂ 10^-3^**
**Etablissement entreprises**			
Relation		0,42	**˂ 10^-3^**
Communication		0,15	0,20
			

+ coefficient de corrélation de Pearson r

**Tableau 4:** Modèle final de régression linéaire multi-variée influençant la satisfaction de carrière

Variables	coefficient de Regression	Erreur Standard	t	p
intercept	-5,965	5,646	-1,057	0,295
Nombre d'entreprises d’exercice (X_1_)	0,067	0,027	2,487	0,016
Score satisfaction de l’organisation du Travail (X_2_)	0,258	0,096	2,683	0,010
Score satisfaction du Ressenti du métier (X_3_)	0,208	0,079	2,643	0,011
Score satisfaction du Salaire (X_4_)	0,316	0,079	4,021	0,000
Score satisfaction de l’information de la règlementation (X_5_)	0,154	0,076	2,029	0,047

Equation du modèle prédictif

**Y _prédit_ = -5,965+0,067 X_1_+0,258 X_2_+0,208 X_3_+0,316 X_4_+0,154 X_5_**

Y_:_ la satisfaction de carrière

## Discussion

La satisfaction au travail chez les médecins est d'une importance capitale pour les médecins, leurs patients et les établissements où ils exercent. Non seulement, elle influence la satisfaction des patients et la qualité des soins fournis, mais en plus, elle a un impact sur le turnover des médecins et le bien être du personnel de la santé. Selon Iellatchitch et Mayrhoffer [7] « La carrière se rapporte à une organisation au sein de laquelle sont ouvertes des opportunités d'ascension progressive de nombreux échelons hiérarchiques, ascension répondant à des règles strictes et préétablies ». La satisfaction vis-à-vis de la carrière constitue selon de nombreux auteurs l'un des déterminants majeurs de la satisfaction au travail [8,9]. De même, Nawaz et Pangil dans leur étude à propos du turnover des salariés, menée en 2015 auprès de 270 salariés, ont objectivé que l'insatisfaction vis-à-vis de la carrière et son évolution constituent l'une des principales causes de l'augmentation du «turnover » des salariés [10]. A notre connaissance et jusqu'à présent peu d'études dans la littérature se sont intéressé à la satisfaction de carrière chez les médecins du travail. Notre étude est la première du genre en Tunisie, où la seule autre étude réalisée à propos de la satisfaction au travail des médecins, a concerné les médecins généralistes [11]. Dans notre étude, 58% des Médecins du Travail de terrain interrogés étaient insatisfaits de leur carrière (58,2%) avec un score global moyen de satisfaction de 43,4±19. Aucun facteur sociodémographique n'a influencé ce score. Nos résultats sont discordants de ceux de la littérature où les études menées chez des médecins toute spécialité confondue rapportent un taux d'insatisfaction de carrière variant de 13 à 30% [12,13]. En fait, nos résultats étaient prévisibles vue la situation actuelle en matière de législation de carrière des médecins du travail. En effet, aucun grade, ni échelon, ni même statut professionnel n'est mis en vigueur pour cette catégorie de médecins en exercice malgré les multiples révisions du décret du 25 octobre 1956 instituant les services de Médecine du Travail dans les entreprises. S'agissant de l'influence des facteurs sociodémographiques sur la satisfaction au travail, plusieurs études ont prouvé l'absence de relation significative ce qui corrobore nos résultats [13,14].

En revanche, l'étude de l'influence de l'âge sur la satisfaction de carrière des médecins au canada, aux Etats-Unis et au Norvège a montré que celui-ci influençait positivement la satisfaction de carrière des médecins canadiens et négativement celle des médecins américains. En revanche, au Norvège il n'y avait aucune influence de l'âge sur la satisfaction de carrière ce qui concorde avec nos résultats [15]. Selon une revue de la littérature pratiquée par Danielle Scheurer et al aux Etats-Unis sur une période de 37 ans allant de 1970 à 2007 et qui a inclut 97 articles, la satisfaction au travail n'a pas été un paramètre statique mais plutôt dynamique. Il aurait été significativement influencé par des facteurs intrinsèques liés au médecin à savoir l'âge et la spécialité et des facteurs extrinsèques liés au travail tels que la demande d'emploi, le contrôle du travail, le soutien des collègues, la capacité à maintenir une bonne relation médecin-patient, l'environnement de travail, la rémunération et les modalités d'encouragement. Ces derniers facteurs constituent une piste d'action sur laquelle on peut agir à fin de contribuer à une meilleure satisfaction au travail [16]. Selon le modèle final prédictif de la satisfaction de carrière des MT des GMT tunisiens, la satisfaction de carrière chez les médecins du travail tunisiens était tributaire d'un plus grand nombre d'entreprises à charge du médecin, d'une meilleure organisation du travail, d'un meilleur ressenti du métier, de la satisfaction du salaire et d'une meilleure information sur la réglementation en vigueur. Ce Modèle expliquait 66,4% de la fluctuation de la satisfaction de carrière. Un nombre d'entreprises à charge important est synonyme d'un enrichissement de l'exercice et de l'expérience professionnelle des médecins, ce qui pourrait expliquer leur plus grande satisfaction en rapport avec cet item.

Par ailleurs, la satisfaction de carrière est aussi significativement dépendante de l'organisation du travail. Quand on aborde l'organisation de l'emploi du temps du médecin de travail, devant se déplacer d'une entreprise à l'autre en respectant une marge horaire bien définie, tenant rarement compte des déplacements ainsi que des problèmes d'organisations internes spécifiques à chaque entreprise, il est tout à fait compréhensible que la satisfaction de carrière des médecins de travail des groupements de médecine de travail tunisiens soit dépendante de l'organisation de travail. En effet, le moindre retard du programme d'activité du médecin peut être responsable d'un décalage horaire qui va empiéter sur l'horaire de pause des ouvriers qui s'abstiennent de consulter durant leur pause. Le médecin de travail est alors obligé d'attendre la reprise de l'activité de l'entreprise une demi-heure à une heure après. De plus des problèmes d'organisation relatifs à la disponibilité des locaux de déroulement des visites médicales dans l'entreprise peuvent entraver l'activité médicale. En effet, même si la législation tunisienne stipule l'obligation de la disponibilité d'un local dédié aux consultations de médecine de travail selon des critères bien définis [17], la réalité de l'exercice est toute autre, et même l'inspection médicale au travail est obligée de faire des concessions dans ce sens au regard de l'activité économique du pays. Nos résultats concordent avec ceux de J.F.Gehanno, qui a mené une étude entre 2002 et 2004 et a objectivé un taux d'insatisfaction au travail chez les internes de médecine du travail en France de l'ordre de 13%. Les principaux motifs d'insatisfaction étaient l'organisation du travail dans les services interentreprises, l'autonomie professionnelle, les perspectives professionnelles (contrats de durée déterminée, médecin remplaçant, absence d'évolution de carrière), la difficulté du monde de l'entreprise, les conditions d'exercice (manque de temps pour les visites d'entreprise et le traitement des dossiers difficiles, kilomètres parcourus, absence de secrétaire) [12].

Au Pays-Bas, Harmen[14], dans une étude où il a exploré l'impact de la commercialisation des services de santé au travail sur la satisfaction de carrière chez les médecins du travail, a montré que les médecins du travail du secteur privé sont plus satisfaits dans leur carrière que ceux appartenant aux structures de santé et sécurité au travail installées au sein de l'entreprise. Ces groupes de médecins du travail considéraient que les conditions du travail étaient désagréables et stressantes [14]. L'insatisfaction de carrière des médecins, résultant des heures de travail dédiées aux tâches administratives a été rapportée dans la littérature [15,18]. Dans un autre registre, une étude Taïwanais menée auprès de 839 médecins toute spécialité confondue, a montré que l'insatisfaction au travail était plutôt en rapport avec l'insatisfaction de la relation médecin-patient et par l'insatisfaction de spécialité [19]. Dans notre étude à l'instar de la littérature scientifique, la satisfaction vis-à-vis du salaire a constitué un déterminant majeur de la satisfaction au travail [2,20–23]. Classiquement, rapportée par la littérature, l'insatisfaction vis-à-vis du salaire constitue une cause majeure du turnover des employés de façon générale et des médecins de façon particulière [24,25]. Dans le domaine médical plusieurs études confrontent ces conclusions. En effet, Stoddard [26] a objectivé dans son étude sur la satisfaction de carrière chez les médecins une relation significative entre satisfaction de carrière et satisfaction de salaire. Une autre étude menée en Arabie saoudite auprès de 344 médecins généralistes et spécialistes dans un hôpital de santé tertiaire, a montré un taux d'insatisfaction au travail de 30%. Le principal facteur significativement associé à l'insatisfaction était le revenu. Les médecins les plus satisfaits de leur revenu étaient les plus satisfaits de leur carrière [13]. Enfin, la satisfaction au travail en relation avec la carrière a été étudiée chez 813 médecins du travail polonais et a conclu que les possibilités limitées de carrière professionnelle, plus souvent rencontrées dans le domaine de la médecine du Travail, que dans d´autres domaines des soins de santé, n'encourageaient pas à entreprendre la spécialisation en médecine du travail [27].

Limites de l'étude :Le questionnaire utilisé « Saphora-Job^®^ » dans sa version 2004 est une échelle mixte qui mesure la satisfaction à travers des indicateurs de satisfaction au travail, validée et adaptée aux spécificités du secteur de la santé. Néanmoins, l'inconvénient majeur pour ce type de questionnaire auto-administré, semble être son taux de réponse assez faible [28–30].Notre taux de réponse était de 52%. Le principal problème semblait provenir de la réticence de certains administrateurs par rapport au concept de l'enquête de peur d'une dévaluation de leur groupement par comparaison aux autres bien que la confidentialité des données ait été garantie au préalable. La principale critique vis-à-vis de l'outil utilisé « Saphora-Job^®^ » nous a semblé l'absence de mesure de l'environnement de travail et de l'épuisement professionnel. En termes de représentativité, la participation des groupements de l'ouest de la Tunisie a été minime. Ce résultat est à nuancer du fait que la répartition des médecins du travail entre les différents groupements est à la base très inégale, les groupements de l'ouest étant les moins peuplés. Un autre facteur de biais est l'absence de spécialistes en médecine de travail dans les groupements de médecine de travail. En effet, une formation de spécialité permet d'avoir le bagage nécessaire pour aborder les problèmes de travail du terrain.

## Conclusion

L'absence de statut professionnel propre aux Médecins du Travail au GMT, garantissant leur indépendance de jugement et d'exercice vis-à-vis des chefs d'entreprises aboutit à d'énormes frustrations et sources d'insatisfaction vis-à-vis de la carrière. Les problèmes organisationnels liés au travail dans les entreprises et un salaire insuffisant en sont aussi responsables. Au terme de ce travail, il nous semble indiqué de réviser la législation tunisienne en matière de Santé et Sécurité au Travail en concertation avec les divers intervenants et de réfléchir à l'intégration des Groupements de Médecine du Travail au sein d'un office étatique sous tutelle du Ministère de la Santé garantissant d'une part une plus grande équité des moyens entre les différentes régions et d'autre part une indépendance d'exercice du Médecin du Travail de première ligne. Il est impératif de prévoir, dans ce contexte, un statut avec progression par échelon pour les médecins de travail des groupements de médecine de travail à l'instar des médecins de la Santé Publique et des médecins de la Caisse Nationale d'Assurance Maladie. A cet effet, l'uniformisation des salaires des médecins de travail dans les 22 groupements de médecine de travail en Tunisie sera ainsi assurée. Par ailleurs, l'intégration dans la formation des médecins spécialistes en médecine de travail tunisiens, une formation pratique obligatoire au sein des groupements de médecine de travail nous semble inévitable. Elle leur permettra d'être plus en contact avec la réalité du terrain, de mieux cerner les contraintes de travail et ainsi de proposer les solutions adéquates pour chaque poste de travail.

### Etat des connaissances actuelle sur le sujet

- La satisfaction au travail chez les Médecins a fait l'objet de nombreux travaux dont les résultats ont montré une grande divergence entre les différents pays;
- Les particularités des conditions de l'exercice de la Médecine de Travail du terrain en Tunisie, fait que le turn over de cette catégorie de Médecins est élevé.

### Contribution de notre étude à la connaissance

- La satisfaction au travail des Médecins de travail des groupements de Médecine de travail tunisiens est tributaire d'une plus grande équité des moyens d'exercice entre les différentes régions et de la garantie de leur indépendance d'exercice;
- Les problèmes organisationnels liés au travail dans les entreprises et le salaire insuffisant sont par ailleurs une grande source d'insatisfaction au travail;
- Le législateur tunisien se doit de réfléchir à l'intégration des Groupements de Médecine du Travail au sein d'un office étatique sous tutelle du Ministère de la Santé.

## Conflits d’intérêts

les auteurs ne déclarent aucun conflit d'intérêts.
